# Potential community and public health impacts of medically supervised safer smoking facilities for crack cocaine users

**DOI:** 10.1186/1477-7517-3-1

**Published:** 2006-01-10

**Authors:** Kate Shannon, Tomiye Ishida, Robert Morgan, Arthur Bear, Megan Oleson, Thomas Kerr, Mark W Tyndall

**Affiliations:** 1 British Columbia Centre for Excellence in HIV/AIDS, Vancouver, Canada; 2 Rock User's Group (RUG) of Vancouver Area Network of Drug Users (VANDU), Vancouver, Canada; 3 Faculty of Medicine, University of British Columbia, Vancouver, Canada

## Abstract

There is growing evidence of the public health and community harms associated with crack cocaine smoking, particularly the risk of blood-borne transmission through non-parenteral routes. In response, community advocates and policy makers in Vancouver, Canada are calling for an exemption from Health Canada to pilot a medically supervised safer smoking facility (SSF) for non-injection drug users (NIDU). Current reluctance on the part of health authorities is likely due to the lack of existing evidence surrounding the extent of related harm and potential uptake of such a facility among NIDUs in this setting. In November 2004, a feasibility study was conducted among 437 crack cocaine smokers. Univariate analyses were conducted to determine associations with willingness to use a SSF and logistic regression was used to adjust for potentially confounding variables (p < 0.05). Variables found to be independently associated with willingness to use a SSF included recent injection drug use (OR = 1.72, 95% CI: 1.09–2.70), having equipment confiscated or broken by police (OR = 1.96, 95% CI: 1.24–2.85), crack bingeing (OR = 2.16, 95% CI: 1.39–3.12), smoking crack in public places (OR = 2.48, 95% CI: 1.65–3.27), borrowing crack pipes (OR = 2.50, 95% CI: 1.86–3.40), and burns/ inhaled brillo due to rushing smoke in public places (OR = 4.37, 95% CI: 2.71–8.64). The results suggest a strong potential for a SSF to reduce the health related harms and address concerns of public order and open drug use among crack cocaine smokers should a facility be implemented in this setting.

## Introduction

Vancouver's Downtown Eastside (DTES) has been the site of an explosive HIV and HCV epidemic associated with a large open illicit drug use scene[1,2]. The health related concerns of injection drug use, particularly blood borne transmission, have been documented extensively [3,4]. In response to these health related risks and concerns of public order in this community, several harm reduction initiatives have been implemented recently and in September 2003 Vancouver received an exemption from Health Canada to pilot the first supervised injection facility in North America [5].

To date, the scientific evaluation of the supervised injection facility has documented several successes including high uptake of the facility, improved public order [6], and a positive impact in reducing syringe sharing locally [7]. However Vancouver is still contending with a open drug scene and issues of public order, particularly among non-injection drug using (NIDU) crack cocaine smokers and crystal methamphetamine users [8]. Growing evidence has highlighted the health related harms of crack cocaine use, including the risk of non parental transmission of HCV, through the sharing of non-injection drug use paraphernalia [9-11], and risky sexual behaviours[12,13]. Crack smokers are known to have a high prevalence of oral lesions, including burns, blisters, and sores, on lips and mouth that may facilitate the oral transmission of blood-borne infections [14]. In addition, crack cocaine smokers have been shown to be at increased likelihood of engaging in high risk sexual and drug related behaviours associated with both HCV and HIV infections [12].

In Europe, several safer smoking facilities (SSF) for non-injection drug users have been opened in addition to supervised injection facilities [15], and yet in North America, the extent and potential uptake of such a facility among NIDUs have not been characterized. Currently supervised drug consumption facilities are operating in 36 cities across four European countries [15]. The existing literature indicates that the ideal drug consumption room is made up of three sections: a clinical area for injecting, a well-ventilated area for free-basing or chasing, and an adjacent common room where no drug use is allowed [16]. Of the 22 drug consumption facilities in the Netherlands, all 22 facilities include spaces for injectors and inhalation areas for crack and heroin smokers [15]. The primary mode of drug consumption in Dutch consumption rooms is smoking or chasing[16], and increasingly present in both German and Swiss facilities [17]. Of the 12 drug consumption facilities in Switzerland, 8 provide spaces for both injection and inhalation. The objectives of a safer smoking facilities are similar to those of injection facilities including a safe environment that enables low-risk, more hygienic drug consumption, reducing the health related risks of drug use and sharing of smoking paraphernalia, minimising the open drug use scene and associated public nuisance, and establishing contact with hard-to-reach drug user populations [18-21]. Through engaging high-risk populations that would otherwise remain outside of conventional medical care, SSF aim to increase potential uptake of health services, drug treatment and addiction services, referral to housing and social supports, and ultimately stabilize and promote client health [15]. Similar to safe injection facilities, SSF provide sterile drug use equipment, a clean and safe environment to use drugs, and education describing the risks of crack smoking and safer ways to smoke. As well, a key opportunity exists at a SSF to delay or prevent the transition from inhaling to injecting drugs [22]. Previous studies have shown that 85% of IDUs engage in illicit non-injection drug use prior to initiating injection drug use [23], and evidence suggests that interventions need to target crack smokers to prevent transition to injecting [24].

Currently advocates and policy makers in Vancouver, Canada are calling for a medically supervised safer smoking facility to smoke pre-obtained non-injection drugs, particularly crack cocaine, and consideration is being given to applying for a federally-administered exemption from Health Canada (under exemption 56) to pilot a safe inhalation room [25,26]. Although evidence of the extent of the health related risks of crack cocaine smoking is growing, the reluctance on the part of health authorities is likely due to the lack of existing evidence of crack use harms and potential uptake of a SSF by crack cocaine smokers in this setting. As such, a partnership between the Rock Users' Group of VANDU and CHASE, a community-based research project, undertook an assessment of the willingness to use a safer smoking facility should one be made available.

## Methods

### Vancouver Area Network of Drug Users (VANDU)

The Vancouver Area Network of Drug Users (VANDU) is a drug user organization that formed in 1997 in response to a growing HIV epidemic and health emergency in the Downtown Eastside of Vancouver associated with illicit drug use, and perceived government inaction. The mission of VANDU is "to improve the lives of people who use illicit drugs through user-based peer support and education"[27]. Today, the organization has grown to include approximately 1500 members and is well known internationally as one of the most organized drug user associations in the world. In addition to ongoing political activism and advocacy, VANDU has expanded over the years to include public education, and peer support and care programs for methadone users and Hepatitis C positive individuals. As well the organisation provides a syringe exchange and recovery program, alley patrol, and street and hotel-based programs.

The Rock Users' Group (RUG) was formed through VANDU in response to a growing need to address the health needs of crack cocaine smokers. The RUG group meets weekly to educate members on the related concerns of crack cocaine use and discuss ways to expand harm reduction initiatives to include crack smokers. Through private donations, RUG has recently begun distributing safer smoking kits to users in the community at a cost of Can$1, including a mouthpiece, durex pipe, brass screens, lubricant, and condoms.

### The Community Health and Safety Evaluation (CHASE) project

The Community Health and Safety Evaluation (CHASE) Project is a prospective open cohort that was established to evaluate the impacts of recently implemented health initiatives on residents of the DTES; to identify priority health issues; shortfalls; and populations at greatest risk. All community residents are eligible to participate, and are enrolled through various recruitment strategies including community-based organizations, several storefront locations and door-to-door initiatives in a single room occupancy hotel (SRO) and subsidised housing buildings. The goal is to enrol a large representative sample of people residing in and having access to services in the DTES community. A short baseline questionnaire is administered by a trained peer interviewer, and elicits questions related to sociodemographic characteristics, health status, service utilization, barriers to healthcare access, and patterns of illicit drug use. In addition, permission is requested to link personal identifiers with a number of health related databases in the province. Participants are followed prospectively through these data linkages on a bi-annual bases. Upon completion of the survey, study participants receive an honorarium of $10 as compensation for their time. University of British Columbia / Providence Health Care Research Ethics Board provided ethical approval for this study.

### Assessment of the health related harms of crack smoking

Through a community-based partnership between the Rock Users' Group (RUG) of Vancouver Area Network of Drug Users (VANDU) and the Community Health and Safety Evaluation (CHASE) Project of the BC Centre for Excellence in HIV/AIDS, the following assessment of health related harms of crack cocaine use was conducted in November of 2004. A total of 437 crack cocaine smokers participated in peer-administered interviews. To be eligible, individuals had to be current crack cocaine smokers (i.e., defined as having smoked crack cocaine in the previous month at the time of interview). Participants were recruited through targeted recruitment strategy that included allocation of referral cards at staggered times and locations over a three-week period. Referral cards were handed-out by CHASE peers through street recruitment and VANDU members on alley patrol and outreach with active crack smokers, as well as through community-based organisations and service providers. Questionnaires were conducted at various storefront locations, and included women's only days to ensure inclusion of multi-risk women. Although participants were asked about former and current injection drug use practices, a history of injection drug use was not considered an eligibility criteria and thus both IDU and NIDU crack smokers were eligible to participate.

Socio-demographic variables that were considered in this analysis included gender, age, ethnicity, housing status, education level, health status, health and addiction service uptake, recent incarceration, and drug use patterns. For the purpose of this analysis, unstable housing was defined as living arrangements that included SRO hotels, transitional housing, and no fixed address/ homelessness. Drug use behaviours included: frequency of cocaine injection, heroin injection, crystal methamphetamine injection, and crack cocaine smoking. As previously [28], "any drug use" was defined as any illicit drug use in the last six months at the time of interview and "frequent drug use" was defined as daily, or most days. Risky drug behaviours included crack bingeing, borrowing crack pipes, smoking in a group of unknown people (such as crack houses, or alleys), and buying used pipes off the street. Public drug use variables included frequency of smoking crack in public places (such as streets, alleys, and parks), having felt in danger when smoking in public places, rushing smoke due to police presence, inhaling brillo / burns due to rushing, and having equipment confiscated or broken by police (without being arrested). Sex work variables included ever having exchanged sex for money or drugs, and having exchanged sex for money or drugs while using crack in the last six months.

Descriptive and univariate analysis were used to determine bivariate associations between willingness to use a SSF and sociodemographic characteristics, selected drug use patterns, crack use behaviours and related risks. Mean averages were used to describe normally distributed variables, and median averages were used to describe skewed variables. Categorical and explanatory variables were analyzed using Pearson X^2^, normally distributed continuous variables were analyzed using t-tests for independent variables, and skewed continuous variables were analyzed using Mann-Whitney U tests. In order to identify factors independently associated with willingness to use a SSF, a logistic regression was performed. Variables found to be associated with willingness to use a SSF at the univariate level (p < 0.05) were entered into the logistic model. All reported p-values are two-sided.

## Results

A total of 437 participants were recruited over a three-week period in November 2004, and thus were eligible for the present analysis. Of the total, 289 (66%) were male and 145 (33%) were female. The median age was 41 years (interquartile range [IQR] = 35 – 45). One-hundred and eighty-four (42%) individuals self identified as Aboriginal, and 335 (77%) were living in unstable housing. Two-hundred and forty-six (56%) reported a history of injection drug use (either former of current IDUs), while 191 (44%) were NIDU crack smokers with no history of injection drug use.

Of the 437 participants, 303 (69%) expressed a willingness to use a medically supervised safer smoking facility (SSF) if one was made available. The univariate analyses of associations between willingness to use a safer smoking facility and sociodemographic characteristics and selected drug use patterns are shown in Table 1. As indicated, willing to use a SSF was positively associated with homelessness (OR = 2.61, 95% CI: 1.19–5.71), having slept outdoors in the last six months (OR = 1.91, 95% CI: 1.22–2.98), exchanging sex for money or drugs while using crack (OR = 1.76, 95% CI: 1.00–3.24), recent IDU (OR = 1.71, 95% CI = 1.27–2.31), injecting with others (OR = 2.15, 95% CI: 1.39–3.32), and injection binge drug use (OR = 2.10, 95% CI: 1.30–3.38).

**Table 1:** Univariate associations between sociodemographic characteristics, selected drug use behaviours and willingness to use a safer smoking facility (SSF)

**Willingness to Use a Safer Smoking Facility**				
**Characteristic**	**Yes**	**No**	**OR**	**p-value**
	**n(%)**	**n(%)**	**(95% CI)**	

**Age**				
Median [IQ range]	40 (35–46)	41 (34–45)		0.729
**Gender**				
Male	199 (67)	90 (68)	0.90 (0.58–1.39)	0.642
Female	103 (34)	42 (32)		
**Ethnicity**				
Aboriginal	136 (45)	48 (36)	1.46 (1.00–2.22)	0.077
Non-Aboriginal	167 (55)	86 (64)		
**Homeless**				
Yes vs. No	43 (14)	8 (6)	2.61 (1.19–5.71)	0.014
**Slept outdoors**				
Yes vs. No	122 (40)	35 (26)	1.91 (1.22–2.98)	0.004
**HIV positive**				
Yes vs. No	81 (27)	30 (22)	1.18 (0.84–1.67)	0.336
**HCV positive**				
Yes vs. No	210 (69)	86 (64)	1.17 (0.90–1.57)	0.290
**Exchanged sex for drugs or money**				
Yes vs. No	107 (35)	38 (28)	1.26 (0.91–1.73)	0.154
**Exchanged sex for drugs or money while using crack (last 6 months)**				
Yes vs. No	55 (18)	15 (11)	1.76 (1.00–3.24)	0.047
**Current IDU**				
Yes vs. No	163 (54)	47 (35)	1.71 (1.27–2.31)	<0.001
**Inject with others**				
Yes vs. No	142 (47)	39 (29)	2.15 (1.39–3.32)	0.001
**Injection bingeing**				
Yes vs. No	108 (36)	28 (21)	2.10 (1.30–3.38)	0.002

The univariate analyses of associations between willingness to use a SSF and crack use behaviours and related risks are shown in Table 2. As indicated, willingness to use a SSF was positively associated with daily crack cocaine use (OR = 1.31, 95% CI: 1.00–1.73), crack bingeing (OR = 2.25, 95% CI: 1.46–3.46), smoking crack in public places (such as, streets, alleys, parks) (OR = 2.59, 95% CI: 1.79–3.12), smoking crack in a group of unknown people (such as crack houses, or alleys) (OR = 2.20, 95% CI = 1.73–5.36), borrowing crack pipes (OR = 2.78, 95% CI: 2.17–3.71), buying used pipes off the street (OR = 2.34, 95% CI: 1.14–4.77), feeling in danger when smoking crack in public places (OR = 2.37, 95% CI: 1.53–3.75), rushed smoking due to police presence (OR = 3.89, 95% CI: 2.44–6.22), inhaling brillo/ burns due to rushing smoke (OR = 4.45, 95% CI:2.55–7.76), and having equipment confiscated or broken by police (without being arrested) (OR = 2.26, 95% CI: 1.48–3.46).

**Table 2:** Univariate associations between crack use behaviours and related risks, and willingness to use a safe smoking facility (SSF)

**Willingness to Use a Safer Smoking Facility**				
**Characteristic**	**Yes**	**No**	**OR**	**p-value**
	**n(%)**	**n(%)**	**(95% CI)**	

**Crack use history**				
≥ 5 years	174 (57)	70 (56)	0.94 (0.62–1.44)	0.786
≥ 10 years	84 (28)	31 (25)	1.11 (0.90–1.57)	0.535
**Daily crack cocaine use**				
Yes vs. No	183 (60)	68 (51)	1.31 (1.00–1.73)	0.060
**Crack bingeing**				
Yes vs. No	228 (75)	77 (58)	2.25 (1.46–3.46)	<0.001
**Smoke crack in public places (ie, streets, alleys, parks)**				
Yes vs. No	170 (56)	53 (40)	2.59 (1.79–3.12)	0.001
**Smoke in a group of unknown people (ie, crack houses, alleys)**				
Yes vs. No	157 (52)	44 (33)	2.20 (1.44–3.36)	<0.001
**Borrowed crack pipes**				
Yes vs. No	212 (70)	76 (57)	2.78 (2.17–3.71)	0.007
**Buy used pipes off the street**				
Yes vs. No	48 (16)	10 (8)	2.34 (1.14–4.77)	0.017
**Cocaine-induced psychosis/paranoia**				
Yes vs. No	116 (38)	45 (33)	1.15 (0.85–1.56)	0.347
**Felt in danger when smoking in public**				
Yes vs. No	139 (46)	35 (26)	2.40 (1.53–3.75)	<0.001
**Rushed smoking due to police presence**				
Yes vs. No	157 (52)	29 (22)	3.89 (2.44–6.22)	<0.001
**Inhaled brillo/burns due to rushing smoke**				
Yes vs. No	121 (40)	12 (9)	4.45 (2.55–7.76)	<0.001
**Equipment confiscated or broken by police (without being arrested)**				
Yes vs. No	159 (53)	44 (33)	2.26 (1.48–3.46)	<0.001

Results of the multivariate logistic regression analysis of factors independently associated with willingness to use a SSF are presented in Table 3. Variables found to be independently associated with willingness to use a SSF included recent injection drug use (OR = 1.72, 95% CI: 1.09–2.70), having equipment confiscated or broken by police (OR = 1.96, 95% CI: 1.24–2.85), crack bingeing (OR = 2.16, 95% CI: 1.39–3.12), smoking crack in public places (OR = 2.48, 95% CI: 1.65–3.27), borrowing crack pipes (OR = 2.50, 95% CI: 1.86–3.40), and inhaled brillo/ burns due to rushing smoke in public places (OR = 4.37, 95% CI: 2.71–8.64).

**Table 3:** Logistic Regression Model of Factors Associated with Willingness to Use a Safer Smoking Facility

**Characteristic**	**AOR**	**95% CI**	**p-value**
	**(95% CI)**		
Current IDU	1.72	1.09–2.70	0.019
Equipment confiscated or broken by police	1.96	1.24–2.85	0.003
Crack bingeing	2.16	1.39–3.12	0.014
Smoking crack in public places	2.48	1.65–3.27	0.002
Borrowing crack pipes	2.50	1.86–3.40	0.006
Inhaled brillo/burns due to rushing smoke	4.37	2.71–8.64	<0.001

## Interpretation

Of a total of 437 crack cocaine smokers, 303 (69%) reported a willingness to use a safer smoking site (SSS) should one be made available. A willingness to use a SSF was associated with recent injection drug use, having equipment confiscated or broken by police, crack binge use, smoking crack in public places, borrowing crack pipes, and burns/ inhaled brillo due to rushed smoking.

The association between crack bingeing and willingness to use a SSF shows a potential for such a facility to intervene in risky drug use behaviours through increased contact and referral with primary care, education and addiction services [29]. Drug bingeing has been previously identified as a high-risk behaviour associated with an elevated risk of HIV seroconversion [30,31]. Intensive crack use has also been associated with increased sexual risk taking, including exchanging sex for drugs or money, multiple sex partners and unprotected sexual encounters[12,13].

In this context, the observed association between borrowing crack pipes and willingness to use a SSF is particularly noteworthy given the increasing evidence of blood borne transmission through the sharing of non-injection drug use implements and the potential of a SSF to reach this high-risk group. Oral sores, cuts, and burns are common among crack cocaine smokers and have been shown to facilitate HCV transmission, as well as increased potential risk for HIV transmission, through the sharing of contaminated equipment, such as crack pipes [9,10,32]. Given the ability of Hepatitis C virus to maintain its infectivity in the environment, the risk of sharing of drug use equipment is particularly concerning [11]. Increasing evidence highlights a higher rate of HCV infection among crack cocaine and heroin smokers reporting no history of injection drug use when compared to the general population[10]. A previous study among female drug users with no history of injection use, found that the sharing of both oral and intranasal non-injection drug use implements was a significant and independent predictor of HCV infection after accounting for other known transmission routes [9]. Given the high prevalence (51%) of individuals having smoked in a group of unknown people (such as crack houses or alleys) in the last six months, as well as the high number of injection drug using crack smokers in this study, the potential for sharing of crack pipes between IDU and NIDU smokers highlights an increased likelihood for infectious disease transmission. In light of these findings, and the previously observed impacts of drug consumption facilities on sharing of drug use equipment [7], SSFs may have the potential to reduce harms associated with crack pipe sharing in this setting.

The observed association between willingness to use a SSF and recent injection use is also particularly relevant, given that this feasibility study was conducted a year following the implementation of the SIF in this community. As mentioned above, the SIF has had positive impacts by improving public order[6], minimising the number of discarded needles in public places, and reducing local syringe sharing [7]. However, the open drug use scene in alleys, doorways and parks persists[8], and is likely reflective of the high rates of NIDU, particularly crack smokers, in this community. As well, given the high percentage of dual users in this study, the implementation of a SSF may help to engage IDU crack smokers who continue to consume drugs in public places. In addition, SSFs also have the opportunity to delay or prevent the transition from inhaling to injecting drugs though prevention-transition programs [22]. Recent studies have suggested that the infection risk hierarchy should be updated to include the public health importance of preventing transition to injection drug use[29], and approximately 85% of IDUs report non-injection drug use prior to initiation into injection use[23].

Given the objective of drug consumption rooms to reduce public nuisance and a visible drug scene, the observed association between public crack use and increased willingness to use a SSF is also noteworthy. Although there is currently limited information available exclusively on SSFs, preliminary findings in Switzerland show an increase in public order and increased contact between NIDUs and health and social services[17]. As well, several studies of drug consumption rooms in Europe have highlighted the benefits of both injection and inhalation areas to increase public order, engage high-risk groups, and reduce visible drug scenes [18-21].

Within the context of public drug use, the associations between willingness to use a SSF, and burns or/ inhaled brillo due to rushing smoke and having equipment confiscated or broken by police, highlight a strong potential to reduce the community harms of public crack use and concerns of public order. Common modes of crack cocaine smoking such as metal pipes are known to cause frequent burns and blisters through excessive heat, while glass and durex pipes frequently splinter causing a smoker's lips to cut [14,33]. In addition, brillo or brass screens commonly used as filters in the pipe stem may break up and be inhaled by the user when the process is rushed. The epidemic of crack cocaine has been associated with heightened violence and crime, as well as exploitation of users, particularly women [12]. In Vancouver, speculations suggest widespread human rights violations on public drug users as part of the police crack down on open drug use scene and concerns of public order [34]. Given that a key objective of drug consumption rooms is to provide a safe place to use pre-obtained illicit drugs and hygienic drug use equipment, these findings highlight a potential to move crack smokers out of alleys and streets, minimize risky crack use, and related harms of rushing in public places should a SSF be implemented in this setting. Similar to safe injection facilities[35], SSF would also provide a key opportunity to couple enforcement and public health efforts as police officers could direct NIDUs on the street to a SSF [22].

Several limitations should be considered. First, this study relied on self-reported information and thus is subject to socially desirable reporting. However previous studies have reported the validity of self-reported information among drug user populations[36]. Second, this study ask participants about the willingness to use a safer smoking facility that does not currently exist and thus participants may have been unsure about the potential use of such a facility. However given that a supervised injection facility has recently been implemented in this setting, and the high rates of injection use among crack cocaine smokers, it is likely that individuals would have been familiar with the concept of a drug consumption site. In addition, similar feasibility studies were conducted prior to the opening of the SIF and were highly predictive of the uptake observed following the opening of the SIF[37,38].

The high reported rate of willingness to use a safer smoking facility (SSF) in this study highlights an important opportunity to connect with a known high-risk drug user population. Given the observed associations between willingness to use a SSF and public drug use and related harms, borrowing of crack pipes, and other risky drug use behaviours, this study identifies a strong potential to reduce community health risks, including infectious disease transmission, and address issues of open drug use and concerns of public order, if a facility was implemented in this setting.
